# Abnormal Cardiac Autonomic Regulation in Mice Lacking ASIC3

**DOI:** 10.1155/2014/709159

**Published:** 2014-04-03

**Authors:** Ching-Feng Cheng, Terry B. J. Kuo, Wei-Nan Chen, Chao-Chieh Lin, Chih-Cheng Chen

**Affiliations:** ^1^Institute of Biomedical Sciences, Academia Sinica, 128 Academia Road, Section 2, Taipei 115, Taiwan; ^2^Department of Medical Research, Tzu Chi General Hospital and Department of Pediatrics, Tzu Chi University, Hualien 970, Taiwan; ^3^Institute of Brain Science, Yang Ming University, Taipei 112, Taiwan; ^4^Taiwan Mouse Clinic-National Comprehensive Mouse Phenotyping and Drug Testing Center, Academia Sinica, Taipei 115, Taiwan

## Abstract

Integration of sympathetic and parasympathetic outflow is essential in maintaining normal cardiac autonomic function. Recent studies demonstrate that acid-sensing ion channel 3 (ASIC3) is a sensitive acid sensor for cardiac ischemia and prolonged mild acidification can open ASIC3 and evoke a sustained inward current that fires action potentials in cardiac sensory neurons. However, the physiological role of ASIC3 in cardiac autonomic regulation is not known. In this study, we elucidate the role of ASIC3 in cardiac autonomic function using *Asic3* ^−/−^ mice. *Asic3* ^−/−^ mice showed normal baseline heart rate and lower blood pressure as compared with their wild-type littermates. Heart rate variability analyses revealed imbalanced autonomic regulation, with decreased sympathetic function. Furthermore, *Asic3* ^−/−^ mice demonstrated a blunted response to isoproterenol-induced cardiac tachycardia and prolonged duration to recover to baseline heart rate. Moreover, quantitative RT-PCR analysis of gene expression in sensory ganglia and heart revealed that no gene compensation for muscarinic acetylcholines receptors and beta-adrenalin receptors were found in *Asic3* ^−/−^ mice. In summary, we unraveled an important role of ASIC3 in regulating cardiac autonomic function, whereby loss of ASIC3 alters the normal physiological response to ischemic stimuli, which reveals new implications for therapy in autonomic nervous system-related cardiovascular diseases.

## 1. Introduction

Acid-sensing ion channel 3 (ASIC3) is the most sensitive acid sensor (pH_0.5_ activation ~6.7) predominantly expressed in the peripheral sensory neurons [1, 2]. It can be activated by low extracellular pH to evoke both transient and sustained inward currents, which can be further enhanced by lactate [3, 4]. ASIC3 has been recognized as not only an acid sensor but also a metabolic sensor for lactic acid acidosis [4, 5]. The physiological significance of ASIC3 is emphasized by sensory neurons expressing ASIC3 or ASIC3-like currents innervating skin, heart, muscle, vessels, and many visceral tissues [5–12]. Because of its specific expression in sensory neurons and its property of proton-evoked sustained current, ASIC3 has attracted much attention in pain studies [13–15].

Apart from the sensitivity to pH drop and lactic acid, the expression of ASIC3 is highly specific to sensory neurons [3, 16]. Since those neurons are composed of major cardiac afferents, ASIC3 may help to transduce angina, the chest pain that accompanies cardiac ischemia [17]. This is supported by studies showing that ASIC3 reproduces the functional features of cardiac ischemia-sensing neurons [7, 18]. During angina, the sympathetic division can further trigger a counterproductive sympathetic reflex that causes stronger and faster contraction in response to insufficient oxygen [19]. Although the balance of autonomic nervous control has been considered a key factor in maintaining normal cardiac function, descriptions of the sensory inputs and reflex regulation of the autonomic nervous system are rare. Recent report also described that overexpression of ASIC3 channel in carotid body glomus cells could activate sympathetic nervous activity and induce prehypertension state in young spontaneously hypertensive rat (SHR) and ASIC3 is one of the major components to form pH-sensitive channel in mouse cardiac dorsal root ganglion neurons, implying a significant role of ASIC3 regulating autonomic function [20, 21]. Accordingly, we sought to further explore the* in vivo* role of ASIC3 in modulating the cardiac autonomic nervous system in conscious mice, since cardiac autonomic function has been suggested to reveal the healthy condition of humans. The spectral analysis in detecting heart rate variability (HRV) through electrocardiography (ECG) has enhanced the study of the sympathetic and parasympathetic function of patients and has been subsequently applied in* in vivo* animal studies [22]. Therefore, we used HRV and ECG* in vivo *analysis of* Asic3* knockout (*Asic3* ^*−/−*^) mice to unravel a role of ASIC3 in regulation of cardiac autonomic function.

## 2. Methods

### 2.1. Animals

Mice were raised in a 12 h light/12 h dark cycle (08:00–20:00 lights on) at 21°C and 40% to 70% humidity. All experiments involved male mice aged 8 to 12 weeks. Telemetric surgery was performed at 12 weeks, and ECG was initiated 1 week after the operation.* Asic3* ^*−/−*^ mice were generated as previously described [23]. Congenic 129 heterozygous (*Asic3* ^*+/−*^) mice were derived by breeding* Asic3* chimera with females of 129S2/SvPasCrl wild types (*Asic3* ^*+/+*^), the same mouse strain as from the embryonic stem cells we used.* Asic3* ^*+/+*^ and* Asic3* ^*−/−*^ mice were offspring of the congenic* Asic3* ^*+/−*^ mice intercross. Mouse genotyping was by PCR. For the* Asic3* ^*−/−*^ allele, we used the primers 5′-ATTCAGGCTGCGCAACTGTT-3′ and 5′-TGTGGTCCCAGGACTTGGTA-3′ and for* Asic3* ^*+/+*^ 5′-CACAGCTCCAGGAGGAGTTGAA-3′ and 5′-CCTTGTGACGAGGTAACAGGTA-3′. The animals were bred and cared for in accordance with the current* Guide for the Use of Laboratory Animals* (National Academy Press, Washington DC). The experimental protocols were approved by the local animal use committee (IACUC, Academia Sinica).

### 2.2. Blood Pressure

Systolic blood pressure and heart rate (HR) in the conscious state were measured by use of a blood pressure monitor for mice and rats, Model MK-2000 (Muromachi Kikai, Tokyo, Japan), according to the manufacturer's instructions. Diastolic blood pressure was calculated from the systolic blood pressure and mean blood pressure.

### 2.3. Animal Preparation for Radiotelemetry Study

For ambulatory long-term ECG analysis in the conscious state, a telemetry transmitter (TA10EA-F20, Data Sciences; St. Paul, MN) was implanted in* Asic3* ^*−/−*^ mice (*N* = 16) and corresponding* Asic3* ^*+/+*^ mice (*N* = 15). Mice were anesthetized with pentobarbital (58.5 mg/kg, given intraperitoneally) and the transmitter was positioned in the abdominal cavity and sutured to the inside of the muscle wall. After surgery, mice were given antibiotics (1% chlortetracycline) and housed individually for recovery for at least 7 days. To allow the mice to habituate to the experimental apparatus, each animal in the home cage was placed in the recording environment for 2 h for at least 2 sequential days before being tested. For each recording, animals in their home cages were first brought to the top of the recording plates for 30 min to allow them to become familiar with the situation. ECG was then performed for 2 h (14:00–16:00) in an isolated room. For induced cardiac ischemia, isoproterenol (1.5 mg/kg) was administered intraperitoneally (*N* = 7 of both genotypes).

### 2.4. HRV Studies in Frequency Domain Measure

ECG signals were converted to digitized signals by use of an analog-to-digital converter. Digital signal processing of the bioelectric signals was modified from our previous algorithm designed for humans and rats [24, 25]. Preprocessing of the ECG signals followed the recommended procedure [26]. In brief, the computer algorithm identified each QRS complex and rejected each ventricular premature complex or noise according to its likelihood in a standard QRS template. Stationary R-R intervals (RR) were resampled and interpolated at a rate of 64 Hz to provide continuity in the time domain. A Hamming window was applied to each time segment to attenuate the leakage effect. Our algorithm then estimated the power density of the spectral components from fast Fourier transform. The resulting power spectrum was corrected for attenuation resulting from sampling and the Hamming window. Each component of the spectrogram was subsequently quantified by the method of integration. Three different frequency domain measures of HRV were computed. Cut-off frequencies for power in the low frequency (LF) and high frequency (HF) ranges were based on those previously reported for mice with 129 backgrounds and defined as 0.25–1.00 Hz and 1.00–6.00 Hz, respectively. Total power (TP) frequency was defined as 0.00–8.5 Hz. LF and HF were also measured in normalized units, which represent the relative value of each power component in proportion to the sum of the LF and HF components. LF, HF, and LF : TP ratio of the RR spectrogram were quantified and logarithmically transformed to correct for the skewness of the distribution [27].

### 2.5. Data Collection and Statistics

Because HR and HRV were highly variable between sleeping and awake states in mice, we only analyzed ECG data from the resting state for baseline activity in a 1 min base. ECG results from minutes 1, 6, 11, 16, and so forth were selected, if no movement occurred. Otherwise, the 1 min data were skipped and next acceptable data was used. At maximum, a total of 10 points were collected from 2 h ECG for HR and HRV in each mouse. For resident-intruder tests, we analyzed ECG data in a 30 s base, except for the period of attack. We collected ECG data from each attack lasting more than 2 s. We used one-way ANOVA for all statistical analysis. Data are presented as means ± SEM. A *P* < 0.05 was considered significant.

### 2.6. Real-Time Quantitative RT-PCR

DRG (C8-T3), nodose ganglia, and hearts were dissected from mice of both genotypes. RNA was isolated from a single ganglion homogenized in a 1.5 mL tube with use of a plastic pestle and lysis buffer and purified by use of the RNeasy mini kit (Qiagen). Heart RNA was isolated with Trizol according to the manufacturer's instructions (Invitrogen). RNA was treated with DNase I (4 U/*μ*g) for 20 min at room temperature. Total RNA of a ganglion (300 ng) or heart (650 ng) was reverse-transcribed by Superscript III RT (Invitrogen). One microliter of derived cDNA (approximately 2 ng) was used as a template for real-time quantitative SYBR Green I PCR carried out in the ABI Prism 7700 Sequence Detection System as the manufacturer's instruction (PE Applied Biosystems). The threshold cycle (C_T_) value indicated the fractional cycle numbers at which the amount of amplified target reached a fixed threshold. The C_T_ values of both the target and the internal reference (GAPDH or cyclophilin) were measured from the same samples, and the expression of the target gene relative to that of GAPDH or cyclophilin was calculated by the comparative C_T_ method, which normalized the expression levels and allowed for calculation of the relative efficiency of the target and reference amplification. Primers used for real-time PCR were as follows: ASIC1a forward 5′-ACAAGGCCAACTTCCGTAGC-3′, reverse 5′-ACTTCCCATACCGCGTGAAG-3′; ASIC1b forward 5′-CCCCATGCTCGGGTTGGATG-3′, reverse 5′-TAGGAGCAATAGAGCAGCATG-3′; ASIC2a forward 5′-AAACCGAAGCAGTTCAGCATGCTG-3′, reverse 5′-GCCATCCTCGCCTGAGTTAAAC-3′; ASIC2b forward 5′-AGACAGTGGTTCCGCAAGCT-3′; ASIC3 forward 5′-TGCTCCAGGAAGAGTTGAATG-3′, and reverse 5′CCTTGTGACGAGGTAACAGGTA-3′; capsaicin receptor (TRPV1) forward 5′-TCTCCACTGGTGTTGAGACG-3′, reverse 5′-GGGTCTTTGAACTCGCTGTC-3′; GAPDH forward 5′-GGAGCCAAACGGGTCATCATCTC-3′, reverse 5′-GAGGGGCCATCCACAGTCTTCT-3′; cholinergic receptor muscarinic 1 (M1) forward 5′-AAGTGGCATTCATCGGGATCAC-3′, reverse 5′-GTTGTTGACTGTCTTGAGCTCTG-3′; cholinergic receptor muscarinic 1 (M2) forward 5′-GTGGACAATTGGCTACTGGCTC-3′, reverse 5′-CCTTGTAGCGCCTATGTTCTTG-3′; cholinergic receptor muscarinic 1 (M4) forward 5′-TGCCATCGAGATCGTACCTG-3′, reverse 5′-AGATTGTCCGAGTCACTTTGCG-3′; adrenergic receptor beta 1 (adrb1) forward 5′-CTACTCGTGGCGCTCATCGTT-3′, reverse 5′-AATCCCATGACCAGATCAGCGCT-3′; adrenergic receptor beta 2 (adrb2) forward 5′-TGGTGCGAGTTCTGGACTTCCA-3′, reverse 5′- GAAGGGCGATGTGATAGCAACA-3′; adrenergic receptor beta 3 (adrb3) forward 5′-CATAACCAACGTGTTCGTGACT-3′, reverse 5′-TGACGTCCACAGTTCGCAACCA-3′; and cyclophilin forward 5′-ACAGGTCCTGGCATCTTGTC-3′, reverse 5′-CATGGCTTCCACAATGTTCA-3′.

### 2.7. Western Blotting

Total proteins (100 *μ*g) were extracted from hearts of adult male mice. Samples were separated by SDS-PAGE (10% acrylamide) and transferred to PVDF membrane. Immunoblotting was performed with antibodies against *β*2 AR (dilution 1 : 100; Santa Cruz Biotechnology), m2-mAChR (1 : 250; Chemicon), and actin (1 : 5000; Sigma). Secondary antibody (1 : 5000) for *β*2 AR and m2-mAChR was anti-rabbit IgG conjugated with horseradish peroxidase (HRP) and that for actin was anti-mouse IgG conjugated with HRP (Chemicon). Bound antibodies were visualized on enhanced chemiluminescence according to the manufacturer's instructions. Antibodies were diluted in 50 mM Tris, pH 7.5, and 0.2 M NaCl containing 1% bovine serum albumin and 0.05% Tween 20.

## 3. Results

### 3.1. *Asic3* ^*−/−*^ Mice Showed Abnormal Autonomic Regulation in Heart Rate Variability and Blood Pressure

**Figure 1:**
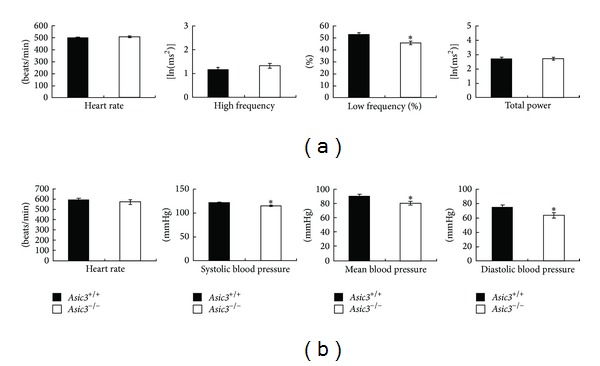
Baseline cardiovascular activity in the* Asic3* ^*−/−*^ mice. (a) Telemetric studies showed both* Asic3* ^*+/+*^ and* Asic3* ^*−/−*^ mice with similar baseline HR in resting status (501 ± 5 bpm versus 507 ± 4 bpm).* Asic3* ^*−/−*^ mice exhibited lower LF% but normal HF and TP as compared with* Asic3* ^*+/+*^ mice (*P < 0.05, *N* = 15 for* Asic3* ^*+/+*^ and *N* = 16 for* Asic3* ^*−/−*^). (b)* Asic3* ^*−/−*^ mice showed significantly lower systolic, mean, diastolic blood pressure than* Asic3* ^*+/+*^ mice (*P < 0.05). The mean HR of conscious* Asic3* ^*−/−*^ mice (*N* = 13) was 570 ± 22 bpm and that of* Asic3* ^*+/+*^ mice (*N* = 13) was 596 ± 17 bpm.

*Asic3* ^*−/−*^ mice had anatomically normal hearts and were viable. To explore the role of ASIC3 in the regulation of autonomic nervous system-related cardiac activity without perturbation by handling or use of anesthetics, we used ambulatory radiotelemetry for ECG recording and HRV study and expressed values as power spectral density. Baseline ECG data were recorded for two consecutive days. We analyzed HR and HRV at resting status on day 2.* Asic3* ^*−/−*^ mice showed normal HR on resting ECG as compared with* Asic3* ^*+/+*^ mice. On HRV analysis,* Asic3* ^*−/−*^ mice showed normal HR and normal HF and TP spectral density as compared with* Asic3* ^*+/+*^ mice (Figure 1(a)). However,* Asic3* ^*−/−*^ mice showed significantly lower ratios in LF power spectral density (LF%) than* Asic3* ^*−/−*^ mice. As compared with the resting status, in the stressed situation, both* Asic3* ^*+/+*^ and* Asic3* ^*−/−*^ mice increased HR during tail-cuff measurement of blood pressure (Figure 1(b)). Despite their increased HR,* Asic3* ^*−/−*^ mice showed significantly lower blood pressure than* Asic3* ^*+/+*^ mice under stress.

### 3.2. *Asic3* ^*−/−*^ Mice Showed Abnormal Response to Isoproterenol-Induced Tachycardia

**Figure 2:**
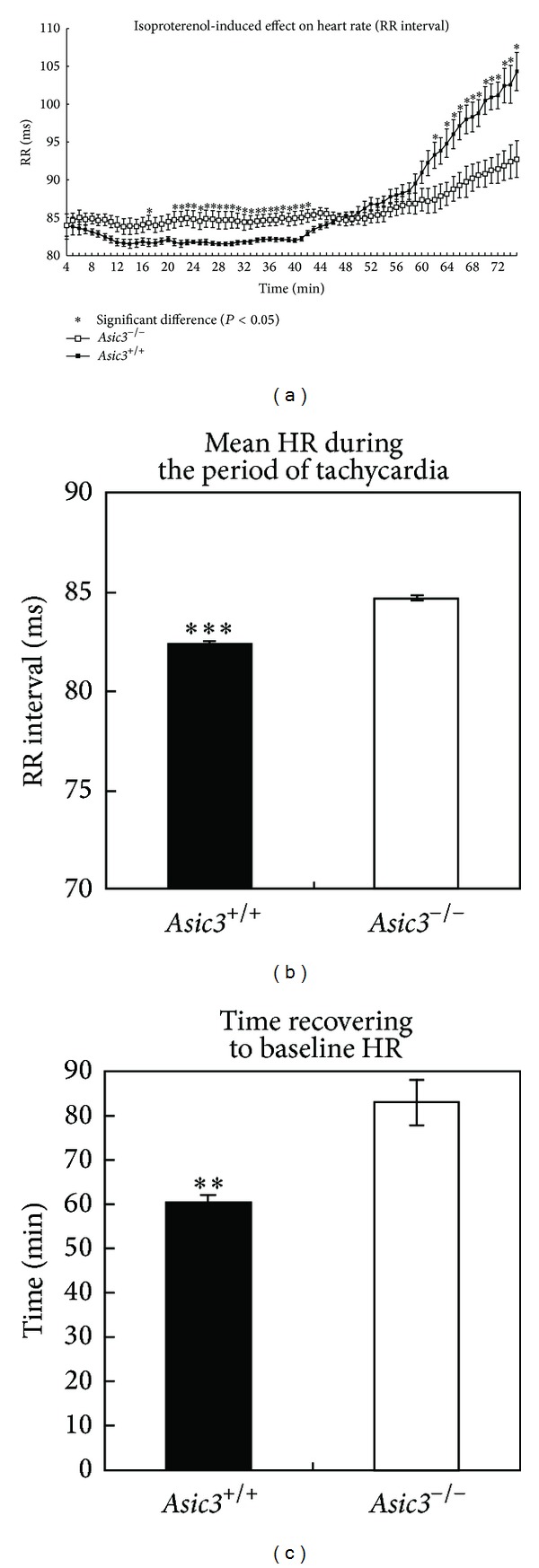
Isoproterenol-induced tachycardia (a) ECG of RR interval showing tachycardia from both genotypes 5 min after isoproterenol injection (1.5 mg/kg given intraperitoneally) on radiotelemetry.* Asic3* ^*+/+*^ mice showed higher HR than* Asic3* ^*−/−*^ mice in most periods of tachycardia (*P < 0.05).* Asic3* ^*−/−*^ mice needed a longer time to slow down the HR to baseline than* Asic3* ^*+/+*^ mice. (b) Mean HR during isoproterenol-induced tachycardia is significantly different between genotypes; tachycardia defined as RR interval less than 90 ms;* Asic3* ^*−/−*^ mice 84.7 ± 0.1 ms versus* Asic3* ^*+/+*^ mice 82.4 ± 0.1 ms. (c) Mean duration of isoproterenol-induced tachycardia of* Asic3* ^*−/−*^ mice 82.9 ± 5.1 min versus* Asic3* ^*+/+*^ mice 59.7 ± 1.7 min (**P < 0.01, ***P < 0.001; *N* = 7* Asic3* ^*+/+*^ mice; *N* = 7* Asic3* ^*−/−*^ mice).

Lastly, we wondered whether loss of ASIC3 had any effect on the myocardium under chemical stimulation such as cardiac ischemia. Isoproterenol is a potent-adrenergic agonist known to induce tachycardia and myocardial ischemia when given in high doses to rodents [28]. Both genotypes produced the expected ECG pattern of sinus tachycardia with a period of ST depression on isoproterenol treatment (Figure 2(a)). However,* Asic3* ^*−/−*^ mice showed a blunted response to isoproterenol-induced tachycardia, with only subtle HR increment (mean RR interval 84.7 ± 0.13 ms for* Asic3* ^*−/−*^ versus 82.4 ± 0.08 ms for* Asic3* ^*+/+*^ mice, Figure 2(b)) but prolonged duration to recover to baseline HR (defined as RR = 90 ms, Figure 2(c)).

### 3.3. Differential Expression of* Asic3* and* Trpv1* Transcripts in DRG and Nodose Ganglia

**Figure 3:**
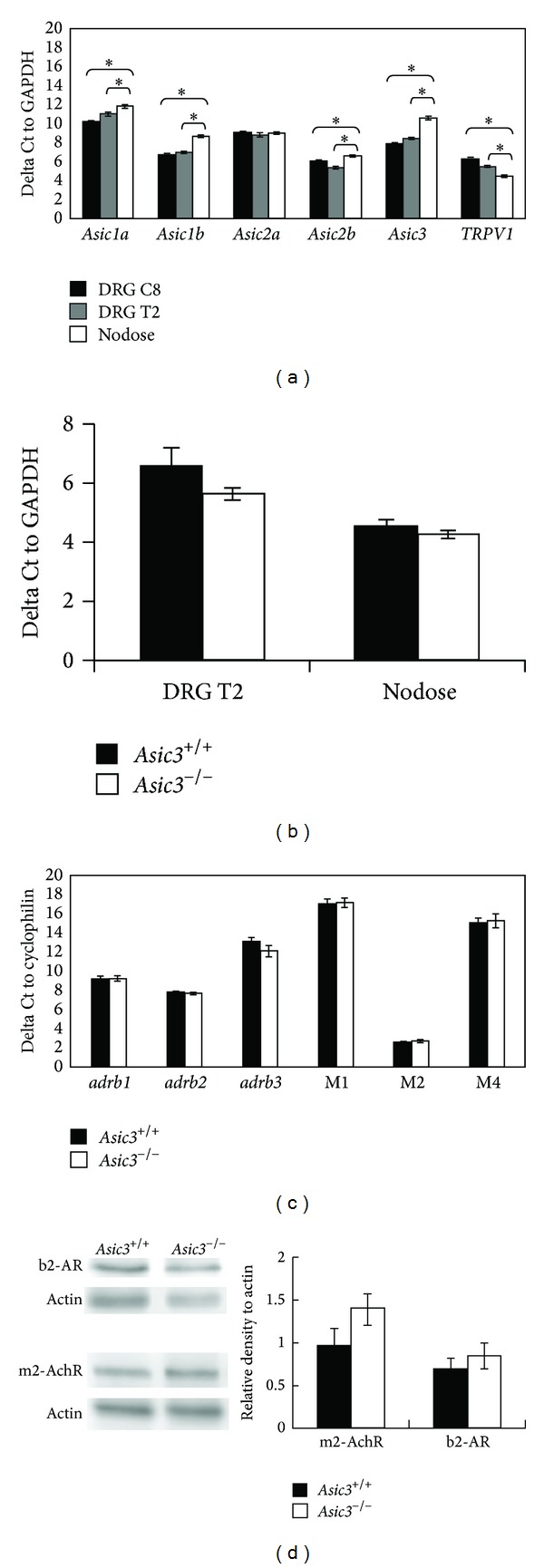
Quantitative RT-PCR and Western blotting analyses of gene expression in sensory ganglia and heart. (a) The expression of* Asic1a*,* Asic1b*,* Asic2b*, and* Asic3* is significantly lower in nodose ganglia (4- to 8-fold, *P < 0.05) than in DRG (C8 and T2), with the expression of* Trpv1* significantly higher in nodose ganglia than in DRG (*P < 0.05). (b) The expression of TRPV1 did not significantly differ between* Asic3* ^*−/−*^ and* Asic3* ^*+/+*^ mice in T2 DRG and nodose ganglia. (c) Gene expression of muscarinic acetylcholine receptors (M1, M2, and M4) and beta-adrenoceptors (*adrb1*,* adrb2*, and* adrb3*) not altered in hearts of* Asic3* ^*−/−*^ mice. (d) Protein levels of adrenoceptors (b2-AR) and acetylcholine receptors (m2-AchR2) not altered in hearts of* Asic3* ^*−/−*^ mice on western analysis. Expression of actin protein was used as an internal control. *N* = 3* Asic3* ^*+/+*^ mice and *N* = 3* Asic3* ^*−/−*^ mice.

We compared the expression of two major peripheral acid-sensing molecules,* Asic3* and* Trpv1*, in sympathetic and parasympathetic sensory ganglia in normal mice. Quantitative RT-PCR revealed DRG (C8 and T2) with a higher level of* Asic3* mRNA (4- to 8-fold) than nodose ganglia (Figure 3(a)). In contrast, the expression of* Trpv1* mRNA in nodose ganglia was higher (2- to 4-fold) than that in DRG. Moreover, the expression of other* Asic* subtypes, such as* Asic1a*,* Asic1b*, and* Asic2b*, was higher in the sympathetic division than in the parasympathetic division. The expression of* Trpv1* in T2 DRG and nodose ganglia did not differ between* Asic3* ^*−/−*^ and* Asic3* ^*+/+*^ mice (Figure 3(b)).

### 3.4. *Asic3* ^*−/−*^ Mice Did Not Show Gene Compensation for Muscarinic Acetylcholine Receptors and Beta-Adrenalin Receptors in Hearts

Previously, Northern blot analysis on* Asic1a*,* Asic1b*,* Asic2*,* Asic4*,* Trpv1*, and* Trpv2* genes was done in* Asic3* ^*−/−*^ mice to determine whether the* Asic3* null mutation interferes with the expression of members of the ASIC and TRPV gene families. Our results indicate that no compensatory changes of other ASIC subtypes and TRPV genes occurred in DRG of* Asic3* ^*−/−*^ mice [23]. We next needed to determine whether gene compensation in the heart accounted for the abnormal cardiac autonomic regulation and isoproterenol response in* Asic3* ^*−/−*^ mice. Because muscarinic acetylcholine receptors and beta-adrenoceptors are responsible for autonomic activity, we compared their expression in the heart of* Asic3* ^*−/−*^ and* Asic3* ^*+/+*^ mice. The expression of* M1*,* M2*, and* M4*, as well as* adrb1*,* adrb2*, and* adrb3*, did not significantly differ between the genotypes (Figure 3(c)). The expression of* M3* and* M5* was not detectable in hearts of* Asic3* ^*−/−*^ or* Asic3* ^*+/+*^ mice. We further verified a similar protein expression of* adrb2* and* M2* in hearts of the two genotypes (Figure 3(d)). Therefore, gene compensation was not found in the* Asic3* ^*−/−*^ mice and these results support our hypothesis that ASIC3 has its unique role in cardiac autonomic regulation.

## 4. Discussion

The null mutation of* Asic3* in mice in our study resulted in imbalanced autonomic regulation with decreased sympathetic function.* Asic3* ^*−/−*^ mice showed subtle cardiovascular changes at baseline condition, with no obviously structural or functional anomaly and only lower ratios of LF power spectral density than* Asic3* ^*+/+*^ mice. However, under stress,* Asic3* ^*−/−*^ mice showed significantly lower blood pressure than* Asic3* ^*+/+*^ mice and a maladaptive autonomic response when an ischemic stress was introduced with injection of isoproterenol. Because deletion of* Asic3* produced no obvious gene compensation of the adrenergic and cholinergic receptors in hearts, the disturbed cardiac function in* Asic3* ^*−/−*^ mice might have a sensory neuron origin, with disrupted interaction between ASIC3 and other proton-sensing channels or receptors [29–32].

The dominant expression of* Asic3* in sympathetic than parasympathetic afferent neurons provides molecular support for the disproportionate autonomic compensation of* Asic3* ^*−/−*^ mice. Our present study also provided results consistent with the rationale that gene expression of ion channels and receptors fundamentally differs between sensory neurons of dorsal root and nodose ganglia [33]. Previous studies show that ASIC3 in sensory afferents is involved in not only acid sensing but also mechanic and metabolic sensing [34–38]. Thus, deletion of* Asic3* would disturb the animal's autonomic regulation during rest (reflecting mechanic and metabolic sensing) and loss of appropriate sympathetic afferent response under external stress (reflecting mechanic, metabolic, and acid sensing). Although ASIC3 has been implicated in modulating pain sensation because of its dominant location in the nociceptive sensory pathway, our studies unraveled an important role for ASIC3 in regulating the cardiac autonomic nervous system through altering inputs from cardiac sympathetic afferents, in addition to the conventional baroreceptors in the great vessels or the chemoreceptors in the carotid body [39].

Sympathetic afferents have larger acid-evoked currents than parasympathetic afferents [17]. Accordingly, our PCR data showed sympathetic sensory neurons with higher* Asic3* expression than parasympathetic sensory neurons (Figure 3(a)), which implies that sympathetic afferents are more sensitive to lactic acid acidosis. During exercise and stress, lactate and ATP are released [40, 41], and these two substances can potentiate ASIC3 [42, 43], as well as adaptive responses [44–47], so it is possible that ASIC3 is involved in adaptive reflexes. Since ASIC3 is mostly present in afferents related to sympathetic (DRG) rather than parasympathetic (nodose ganglia) stimulation, this finding agrees with the results of less cardiovascular sympathetic stimulation in* Asic3* ^*−/−*^ mice.

A previous study has shown that ASIC2 also contributes to autonomic regulation. Lu et al. reported that conscious* Asic2* null (*Asic2* ^*−/−*^) mice developed hypertension and a decreased gain of the baroreflex [48]. A predominance of ASIC2 expression over other ASIC subtypes in nodose ganglia was also reported. In contrast to* Asic3* ^*−/−*^ mice, which is presenting a phenotype of decreased sympathetic function,* Asic2* ^*−/−*^ mice had exaggerated sympathetic and depressed parasympathetic control of the circulation. Accumulating evidence has shown that ASIC2 assembles with ASIC3 to form functional heteromeric channels, especially in cardiac sensory neurons [21, 29, 48]. Besides, our previous study also suggests that ASIC3 of nodose ganglia plays a role in low-threshold baroreceptor in regulating blood volume homeostasis [36]. Therefore, deleting either ASIC2 or ASIC3 will disturb the assembling of heteromeric ASIC2/3 channel, which might play a role in autonomic regulation [38].

## 5. Conclusions

We provided an* in vivo* evidence for ASIC3 in regulating cardiac autonomic function, whereby loss of ASIC3 alters the normal physiological response to ischemic stimuli, which reveals new implications for therapy in autonomic nervous system-related cardiovascular diseases. A previous clinical study also described poorer long-term outcomes for patients with silent myocardial ischemia than for those with symptomatic ischemia during dobutamine stress echocardiography [49]; implying loss of normal cardiac sensory input, such as ASIC3 or TRPV1, may have important clinical implication. Therefore, future studies involving* Asic3* ^*−/−*^ mice as a model for acid-evoked reflex loop during angina would provide valuable and informative clues to the therapeutic strategies for patients with cardiac ischemia.

## References

[1] Wu W-L, Cheng C-F, Sun W-H, Wong C-W, Chen C-C (2012). Targeting ASIC3 for pain, anxiety, and insulin resistance. *Pharmacology and Therapeutics*.

[2] Lingueglia E, Lazdunski M (2013). Pharmacology of ASIC channels. *WIREs Membrane Transport and Signaling*.

[3] Waldmann R, Bassilana F, de Weille J, Champigny G, Heurteaux C, Lazdunski M (1997). Molecular cloning of a non-inactivating proton-gated Na^+^ channel specific for sensory neurons. *Journal of Biological Chemistry*.

[4] Immke DC, McCleskey EW (2001). Lactate enhances the acid-sensing Na^+^ channel on ischemia-sensing neurons. *Nature Neuroscience*.

[5] Molliver DC, Immke DC, Fierro L, Paré M, Rice FL, McCleskey EC (2005). ASIC3, an acid-sensing ion channel, is expressed in metaboreceptive sensory neurons. *Molecular Pain*.

[6] Price MP, McIlwrath SL, Xie J (2001). The DRASIC cation channel contributes to the detection of cutaneous touch and acid stimuli in mice. *Neuron*.

[7] Sutherland SP, Benson CJ, Adelman JP, McCleskey EW (2001). Acid-sensing ion channel 3 matches the acid-gated current in cardiac ischemia-sensing neurons. *Proceedings of the National Academy of Sciences of the United States of America*.

[8] Jones NG, Slater R, Cadiou H, McNaughton P, McMahon SB (2004). Acid-induced pain and its modulation in humans. *Journal of Neuroscience*.

[9] Sugiura T, Dang K, Lamb K, Bielefeldt K, Gebhart GF (2005). Acid-sensing properties in rat gastric sensory neurons from normal and ulcerated stomach. *Journal of Neuroscience*.

[10] Hughes PA, Brierley SM, Young RL, Blackshaw LA (2007). Localization and comparative analysis of acid-sensing ion channel (ASIC1, 2, and 3) mRNA expression in mouse colonic sensory neurons within thoracolumbar dorsal root ganglia. *Journal of Comparative Neurology*.

[11] Holzer P (2007). Taste receptors in the gastrointestinal tract. V. Acid sensing in the gastrointestinal tract. *The American Journal of Physiology—Gastrointestinal and Liver Physiology*.

[12] Deval E, Noël J, Lay N (2008). ASIC3, a sensor of acidic and primary inflammatory pain. *The EMBO Journal*.

[13] Sluka KA, Price MP, Breese NM, Stucky CL, Wemmie JA, Welsh MJ (2003). Chronic hyperalgesia induced by repeated acid injections in muscle is abolished by the loss of ASIC3, but not ASIC1. *Pain*.

[14] Yen Y-T, Tu P-H, Chen C-J, Lin Y-W, Hsieh S-T, Chen C-C (2009). Role of acid-sensing ion channel 3 in sub-acute-phase inflammation. *Molecular Pain*.

[15] Cheng C-F, Chen I-L, Cheng M-H (2011). Acid-sensing ion channel 3, but not capsaicin receptor TRPV1, plays a protective role in isoproterenol-induced myocardial ischemia in mice. *Circulation Journal*.

[16] Chen C-C, England S, Akopian AN, Wood JN (1998). A sensory neuron-specific, proton-gated ion channel. *Proceedings of the National Academy of Sciences of the United States of America*.

[17] Benson CJ, Eckert SP, McCleskey EW (1999). Acid-evoked currents in cardiac sensory neurons: a possible mediator of myocardial ischemic sensation. *Circulation Research*.

[18] Yagi J, Wenk HN, Naves LA, McCleskey EW (2006). Sustained currents through ASIC3 ion channels at the modest pH changes that occur during myocardial ischemia. *Circulation Research*.

[19] Armour JA (1999). Myocardial ischaemia and the cardiac nervous system. *Cardiovascular Research*.

[20] Tan Z-Y, Lu Y, Whiteis CA (2010). Chemoreceptor hypersensitivity, sympathetic excitation, and overexpression of ASIC and TASK channels before the onset of hypertension in SHR. *Circulation Research*.

[21] Hattori T, Chen J, Harding AMS (2009). ASIC2a and ASIC3 heteromultimerize to form ph-sensitive channels in mouse cardiac dorsal root ganglia neurons. *Circulation Research*.

[22] Schwartz PJ, Pagani M, Lombardi F, Malliani A, Brown AM (1973). A cardiocardiac sympathovagal reflex in the cat. *Circulation Research*.

[23] Chen C-C, Zimmer A, Sun W-H, Hall J, Brownstein MJ, Zimmer A (2002). A role for ASIC3 in the modulation of high-intensity pain stimuli. *Proceedings of the National Academy of Sciences of the United States of America*.

[24] Yang CCH, Kuo TBJ (1999). Assessment of cardiac sympathetic regulation by respiratory-related arterial pressure variability in the rat. *The Journal of Physiology*.

[25] Kuo TBJ, Yang CCH (2005). Sleep-related changes in cardiovascular neural regulation in spontaneously hypertensive rats. *Circulation*.

[26] Task Force of European Society of Cardiology and the North American Society of Pacing Electrophysiology (1996). Heart rate variability: standards of measurement, physiological interpretation and clinical use. *Circulation*.

[27] Japundzic N, Grichois M-L, Zitoun P, Laude D, Elghozi J-L (1990). Spectral analysis of blood pressure and heart rate in conscious rats: effects of autonomic blockers. *Journal of the Autonomic Nervous System*.

[28] Desai KH, Sato R, Schauble E, Barsh GS, Kobilka BK, Bernstein D (1997). Cardiovascular indexes in the mouse at rest and with exercise: new tools to study models of cardiac disease. *The American Journal of Physiology—Heart and Circulatory Physiology*.

[29] Benson CJ, Xie J, Wemmie JA, Price MP, Henss JM, Welsh MJ (2002). Heteromultimers of DEG/ENaC subunits form H^+^-gated channels in mouse sensory neurons. *Proceedings of the National Academy of Sciences of the United States of America*.

[30] Xie J, Price MP, Berger AL, Welsh MJ (2002). DRASIC contributes to pH-gated currents in large dorsal root ganglion sensory neurons by forming heteromultimeric channels. *Journal of Neurophysiology*.

[31] Huang C-W, Tzeng J-N, Chen Y-J, Tsai W-F, Chen C-C, Sun W-H (2007). Nociceptors of dorsal root ganglion express proton-sensing G-protein-coupled receptors. *Molecular and Cellular Neuroscience*.

[32] Lin Y-W, Min M-Y, Lin C-C (2008). Identification and characterization of a subset of mouse sensory neurons that express acid-sensing ion channel 3. *Neuroscience*.

[33] Peeters PJ, Aerssens J, de Hoogt R (2006). Molecular profiling of murine sensory neurons in the nodose and dorsal root ganglia labeled from the peritoneal cavity. *Physiological Genomics*.

[34] Jones RCW, Xu L, Gebhart GF (2005). The mechanosensitivity of mouse colon afferent fibers and their sensitization by inflammatory mediators require transient receptor potential vanilloid 1 and acid-sensing ion channel 3. *Journal of Neuroscience*.

[35] Page AJ, Brierley SM, Martin CM (2005). Different contributions of ASIC channels 1a, 2, and 3 in gastrointestinal mechanosensory function. *Gut*.

[36] Lee C-H, Sun SH, Lin S-H, Chen C-C (2011). Role of the acid-sensing ion channel 3 in blood volume control. *Circulation Journal*.

[37] Abboud FM (2010). In search of autonomic balance: the good, the bad, and the ugly. *The American Journal of Physiology—Regulatory Integrative and Comparative Physiology*.

[38] Chen CC, Wong CW (2013). Neurosensory mechanotransduction through acid-sensing ion channels. *Journal of Cellular and Molecular Medicine*.

[39] Tan Z-Y, Lu Y, Whiteis CA, Benson CJ, Chapleau MW, Abboud FM (2007). Acid-sensing ion channels contribute to transduction of extracellular acidosis in rat carotid body glomus cells. *Circulation Research*.

[40] Lutmer RF, Wexler BC (1971). Myocardial and serum lactate changes during isoproterenol-induced infarction. *The American Heart Journal*.

[41] Wang Y, Li G, Liang S (2008). Role of P2X_3_ receptor in myocardial ischemia injury and nociceptive sensory transmission. *Autonomic Neuroscience: Basic and Clinical*.

[42] Naves LA, McCleskey EW (2005). An acid-sensing ion channel that detects ischemic pain. *Brazilian Journal of Medical and Biological Research*.

[43] Birdsong WT, Fierro L, Williams FG (2010). Sensing muscle ischemia: coincident detection of acid and ATP via interplay of two ion channels. *Neuron*.

[44] Philp A, Macdonald AL, Watt PW (2005). Lactate—a signal coordinating cell and systemic function. *Journal of Experimental Biology*.

[45] Fattor JA, Miller BF, Jacobs KA, Brooks GA (2005). Catecholamine response is attenuated during moderate-intensity exercise in response to the ‘lactate clamp’. *The American Journal of Physiology—Endocrinology and Metabolism*.

[46] Pan H-L, Longhurst JC, Eisenach JC, Chen S-R (1999). Role of protons in activation of cardiac sympathetic C-fibre afferents during ischaemia in cats. *The Journal of Physiology*.

[47] Liu J, Gao Z, Li J (2010). Femoral artery occlusion increases expression of ASIC3 in dorsal root ganglion neurons. *The American Journal of Physiology—Heart and Circulatory Physiology*.

[48] Lu Y, Ma X, Sabharwal R (2009). The ion channel ASIC2 Is required for baroreceptor and autonomic control of the circulation. *Neuron*.

[49] Biagini E, Schinkel AFL, Bax JJ (2005). Long term outcome in patients with silent versus symptomatic ischaemia during dobutamine stress echocardiography. *Heart*.

